# Use of programme budgeting and marginal analysis as a framework for resource reallocation in respiratory care in North Wales, UK

**DOI:** 10.1093/pubmed/fdv128

**Published:** 2016-10-17

**Authors:** J.M. Charles, G. Brown, K. Thomas, F. Johnstone, V. Vandenblink, B. Pethers, A. Jones, R.T. Edwards

**Affiliations:** 1 Centre for Health Economics & Medicines Evaluation, Bangor University, Ardudwy, Normal Site, Bangor, GwyneddLL57 2PZ, UK; 2 Public Health Wales, Preswylfa. Hendy Road, Mold, Flintshire CH7 1PZ, UK; 3 Public Health Wales, Unit 10, Llys Castan, Parc Menai, Bangor, Gwynedd LL57 4DF, UK; 4 Betsi Cadwaladr University Health Board, Ysbyty Gwynedd, Penrhosgarnedd, Bangor, Gwynedd LL57 2PW, UK

**Keywords:** disinvestment, health economics, healthcare resources, investment, marginal analysis, programme budgeting, public health, resource reallocation

## Abstract

**Background:**

Since the global financial crisis, UK NHS spending has reduced considerably. Respiratory care is a large cost driver for Betsi Cadwaladr University Health Board, the largest health board in Wales. Under the remit of ‘prudent healthcare’ championed by the Welsh Health Minister, a Programme Budgeting Marginal Analysis (PBMA) of the North Wales respiratory care pathway was conducted.

**Methods:**

A PBMA panel of directors of medicines management, therapies finance, planning, public health and healthcare professionals used electronic voting to establish criteria for decision-making and vote on candidate interventions in which to disinvest and invest.

**Results:**

A sum of £86.9 million was spent on respiratory care in 2012–13. Following extensive discussion of 13 proposed candidate interventions facilitated by a chairperson, 4 candidates received recommendations to disinvest, 7 to invest and 2 to maintain current activity. Marginal analysis prioritized mucolytics and high antibiotic prescribing as areas for disinvestment, and medicines waste management and pulmonary rehabilitation for investment.

**Conclusions:**

This exercise demonstrates the potential for health boards to use evidence-based approaches to reach potentially controversial disinvestment and investment decisions. Initial progress has begun with communication from the Medical Director in relation to the disinvestment in mucolytics prescribing and possible redirection of funding options being explored.

## Background

### Respiratory care in North Wales

Since the global financial crisis, NHS spending in the UK has been cut per year by 0.4% on average, leading to a decrease in NHS spending of 7.3% as a percentage of gross domestic product.^1^ The Kings Fund has estimated that a 4% increase in UK productivity is required to close the gap between healthcare funding and need.^2^ A report by The Nuffield Trust comparing healthcare services across the UK found no major differences between the four nations, each nation had made improvements to their NHS during the 2000s.^3^ However, the report did demonstrate that while all four nations reduced healthcare spending since 2010, Wales reduced the budget in real terms, with spending reduced by 4.3% between 2009–10 and 2012–13.^3^ However, ministers have stated they will increase NHS spending in 2014–15 and 2015–16. At this time of culture shift in the NHS in Wales, the Health Minister for Wales, Professor Mark Drakeford, has called 2014 the ‘Year of prudent healthcare’. He has described prudent healthcare as: ‘healthcare that fits the needs and circumstances of patients and actively avoids wasteful care that is not to the patients benefit’.^4^ It is evitable that ministers face difficult decisions regarding healthcare spending and resources. As part of ‘prudent healthcare’ in Wales, this paper describes Programme Budgeting and Marginal Analysis (PBMA) as an approach to make resource allocation decisions that impact the respiratory care pathway in Betsi Cadwaladr University Health Board (BCUHB), the largest health board in Wales.

PBMA is a framework that helps decision-makers to reallocate resources so that benefits are maximized.^5^ PBMA has eight stages: choose a set of meaningful programmes; identify current activity and expenditure in those programmes; think of improvements; weigh up incremental costs and incremental benefits and prioritize a list; consult widely; decide on changes; effect the changes and evaluate progress.^5^

The role of PBMA as a framework for rational disinvestment has been championed by Donaldson *et al*.^6^ PBMA has been used previously as an approach to make resource reallocation decisions in healthcare settings.^7–10^ Bolaji^11^ using PBMA in Lincolnshire UK to assess chronic obstructive pulmonary disease (COPD) and asthma pathways identified disinvestment opportunities, including medicines management and inappropriate access to oxygen therapy. The process also identified investment areas including, tobacco control, non-invasive ventilation and a new pulmonary rehabilitation programme with a focus on patient education. Below, we describe applying PBMA to the respiratory care pathway at BCUHB.

## Methods

### The PBMA operational group

A PBMA operational group composed of a chairperson, BCUHB finance staff, BCUHB and Public Health Wales staff with an interest in respiratory care and health economists with previous experience conducting PBMA exercises. This operational group oversaw the running of the PBMA exercise as a whole, gathering evidence on proposed candidates, arranging meetings and inviting staff to establish the PBMA panel.

Members of the operational group had previous experience undertaking PBMA exercises.^12^ However, we developed new methods during this exercise; these methods are described in more detail below.

### The PBMA panel

The PBMA panel are responsible for making the investment and disinvestment recommendations. For this particular PBMA exercise, we took advice from the Executive Director of Public Health of BCUHB and a project team of BCUHB staff who had undertaken previous work examining the pathway as to whom to invite to the PBMA panel. A PBMA panel was established with representation invited from clinicians (primary and secondary care), nursing staff (primary and secondary care), budget holding healthcare managers (therapies, medicines management, Primary, Community and Specialist Medicine), finance, service users and business support. The panel were invited to take part in the PBMA exercise via e-mail and as their participation was voluntary they were informed they could withdraw their participation from the process at any time. As the exercise was instigated from BCHUB with sponsorship from the Executive Director of Public Health, ethic approval was not required.

**Fig. 1:**
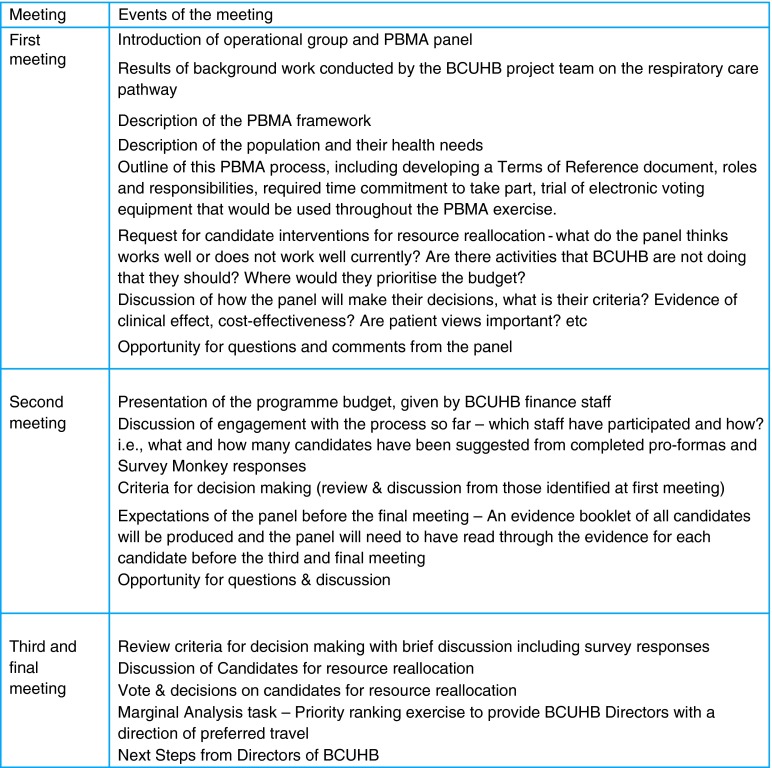
Description of PBMA meeting activities.

The panel met on three occasions between February and June 2014. Meetings were held in accessible locations to minimize travel as much as possible. Video conferencing was also provided at all meetings. These meetings outlined the process, the panel's roles and responsibilities, established and discussed criteria for decision-making and provided an opportunity to discuss and vote on candidate interventions in which to disinvest and invest. A description of what happened during each meeting is described in Fig. 1.

### The programme budget

BCUHB finance staff were asked to provide detail of the 2012–13 spend on respiratory care in BCUHB. Finance staff were also asked to describe this spend in terms of the split between primary and secondary care and spend per head.

### Candidate interventions for resource reallocation

Candidate interventions for resource reallocation were generated by asking for opinions from staff and managers about what they felt worked or did not work in the pathway based on their knowledge and experiences. Candidate interventions were developed through three approaches. First, by initial work conducted by the respiratory project team at BCUHB who critically appraised the pathway, looking to evidence, guidance and other healthcare systems for ways to improve the pathway. Second, based on previous experience,^12^ the panel members were encouraged to submit candidates based on their views or experiences via a pro forma sheet (see Supplementary data, Additional File 1 for example pro forma sheet). This pro forma detailed the types of information required for each candidate and provided an example to ensure the submitted candidates had the same degree of information for ease of comparability and the respondents understood the type and level of information required. As clinicians were involved in the process, we were concerned some would view the pro forma as a time-consuming task; therefore, we decided to improve engagement further by using a third and final approach. An online Survey Monkey questionnaire was created, which divided the pro forma into separate questions. The link to this survey was distributed to all invited panel members who were also asked to forward it onto anyone else who wished to contribute. We asked for wider distribution to increase our chances of developing a broad range of candidates for resource reallocation as part of this consultation exercise.

### Evidence gathering

For each candidate, a rapid review of national guidance included guidance from NICE, British Thoracic Society, IMPRESS and the Cochrane Library of Systematic Reviews. Local guidelines for BCUHB were also used as an evidence source. Where there was limited or no guidance available, a search using key terms for each candidate was conducted using the OVID health database. BCUHB finance staff who were part of the PBMA operational group were also asked to provide information on the potential cost-savings from proposed candidate interventions, where possible, to allow the panel to consider the concept of opportunity cost when appraising the candidate interventions. Opportunity cost is the value of benefits foregone by not using resources in their next best alternative use. All evidence was summarized in an evidence booklet (see Supplementary data, Additional File 2 for an example of the evidence booklet presented to panel members). The evidence booklet contained a description of the proposed candidate; the life course stage the candidate related to; the current spend on this candidate (if the candidate was a service or programme currently conducted by BCUHB), its evidence; potential risks; likely impacts (including cost-savings, where possible) and additional notes for example, relevant reports or points to consider. The booklet was sent to all panel members 2 weeks prior to the final meeting.

### Marginal analysis

A high-level priority ranking task was developed to show a direction of travel to directors of BCUHB, based on this PBMA process. The task used ranking exercises to show the panel's top priority candidates for investment and then disinvestment. This is considered a high-level task as it indicates clear priorities to the board of directors at BCUHB resulting from this exercise.

## Results

### The programme budget

Respiratory illness is a large cost driver for BCUHB. In 2011–12, BCUHB spent £122 per year per head for a population circa 700 000, compared with the Welsh average of £116 per head (age adjusted).^13^ In 2012–13, BCUHB spent £86.9 million on respiratory care with, £61.2 million of spend was accrued by secondary care and £25.7 million by primary care.^13^

### Criteria for appraising candidate interventions

During the first PBMA session, the 16 panel members who were in attendance were presented with the following criteria: evidence of cost-effectiveness; evidence of clinical effectiveness; evidence of impact for reducing inequalities in health; patient views and local experts (healthcare professionals) opinions and views. These criteria were developed by the operational group based on previous PBMA exercises.^12^

Results from an electronic voting exercise demonstrated the panel felt that evidence of clinical effectiveness (*n* = 5), cost-effectiveness (*n* = 8) and impact on reducing health inequalities (*n* = 3) were the most important. The panel were asked whether they wished to suggest any further criteria. The panel suggested one other criterion of effecting changes that were relatively simple and easy to implement so they could be done quickly.

Survey Monkey received the highest response rate with 16 responses; we received 5 pro formas and 3 candidates were generated from the previous work undertaken by BCUHB. We collated all these responses, removing duplicate candidates where necessary. Based on the three approaches of candidate generation (project team work, pro formas and Survey Monkey responses), 13 proposed candidate interventions were generated for discussion by the panel. Each candidate was an individual proposal for resource reallocation, ranging from specific interventions (e.g. pulmonary rehabilitation), to cross agency partnership and working (e.g. establishing links with housing mangers in local authorities to prevent damp, mould and poor insulation in the homes of patients with respiratory illness as these can exacerbate their condition).

### Discussion of candidate interventions

Based upon the three approaches of candidate generation (project team work, pro formas and Survey Monkey responses), a list of 13 candidate interventions was generated. During the final PBMA session, the panel discussed each of the 13 candidate interventions in turn, and voted upon whether to maintain current activity or to seek alternatives by investing or disinvesting in the candidate intervention. At this final session, the panel unfortunately did not have representation from primary care clinicians or patients. However, the panel had representation from medical consultants, nursing staff, pharmacy, public health, informatics support, managers including chiefs of staff and executives. A summary of the discussion and results from the electronic voting by the 13 panel members is presented in Table 1.

**Table 1:** Summary of discussion and the voting exercise results by the 13 panel members after discussion.

*Candidate*	*Number of votes to maintain current activity*	*Number of votes to disinvest*	*Number of votes to invest*
Hospital ward/bay closure If BCUHB invested in more preventative services or services that reduced admissions, we may have scope to reduce the number of inpatient beds potentially saving £300 000 pounds annually. The panel wished the vote to be set within the caveat that if other services were in place, i.e. no backlog of patients and the whole pathway was improved, then they wished to reduce the number of beds on wards. However, it is worth noting one member wished to abstain in the vote as they felt they could not vote based on the current service.	3	9	0
Outpatient follow-up The PBMA process identified that the health board spends a considerable proportion of the budget on outpatient follow-up attributed to a new to follow-up ratio, which is higher than other areas. If the current follow-up ratio was modified in accordance with guidelines available,^14^ resources could be released from outpatient services. The panel wished to improve the current outpatient follow-up ratio by following guidelines.	2	11	0
Misdiagnosis This particularly relates to misdiagnosis around COPD. If we used spirometry, it would reduce the risk of mislabelling patients as having COPD and prescribing them expensive medicines, which may not be in the patient's best interest.^15^ The panel voted unanimously to invest in spirometry training and provision to reduce the level of potential misdiagnosis in respiratory illness.	0	0	13
Skills mix This candidate explores whether the health board could take a holistic approach to caring for patients with multiple morbidities, particularly in terms of outpatient appointments. If a patient attending to see a respiratory specialist, as well as other specialists (e.g. cardiologist and endocrinologist) whether there is potential within the health board to have the patient only see one care of the elderly clinician or a generalist who would co-ordinate those appointments. This could reduce the impact of multiple appointments on the patient first and also the costs associated with that. There is also scope to explore whether staff time could be utilized in different ways, namely using nurse practitioner time or other staff to free up time for the specialists, making the process more efficient. The panel voted to invest in skills mix by following Royal College of Physicians guidelines seeking to recruit more generalists rather than specialists .^16^	2	0	11
High-cost antibiotic prescribing This candidate proposes that BCUHB guidelines are followed for community acquired pneumonia; clinically assessing patients and using only oral medication for patients who have a lower severity score and reserving the higher cost intravenous antibiotics for whom it is indicated. The panel voted unanimously to reduce high-cost antibiotic prescribing by conducting regular audits and engaging health protection colleagues to reduce the level of unnecessary high-cost antibiotic prescribing in the health board.	0	13	0
Medicines waste management Health board audits have demonstrated large levels of medicine waste namely inhalers. Using strategies such as improved prescribing and the use of spirometry, the health board could reduce waste considerably. The panel voted unanimously to reduce medicine waste by tackling this issue in practices, using patient campaigns and targeting new patients to reduce the level of medicine waste in the health board.	0	0	13
Mucolytics Mucolytics are medicines mainly used in the treatment of COPD to reduce sputum viscosity; however, they have a very limited evidence base, and are not indicated as a first-line treatment. BCUHB's spend on mucolytics is quite considerable and the use of these medicines could be reduced by following guidelines, thus reducing subsequent costs. The panel voted unanimously to disinvest in mucolytics by removing it from the formulary, using IT systems to display guidance messages or liaising with Hearts and Minds colleagues to reduce the repeated use of mucolytics in the health board.	0	13	0
Advanced care planning This candidate proposes advanced care planning in the treatment of patients with chronic conditions. The health board could be preparing people earlier of what to expect and what will happen. This may reduce admissions and re-direct admissions particularly in cases where it is not in the patient's best interests to be directed to the Emergency Department if they can be treated better elsewhere for example, in a community hospital, hospice or at home. The panel voted to increase resources such as improving and broadening community care so patients and their families feel confident and safe to be managed at home or in the community.	1	0	12
Pulmonary rehabilitation programme Pulmonary rehabilitation has a strong evidence base with regard to reduced mortality, inpatient days and readmission rates.^17,18^ Pulmonary rehabilitation is also considered cost-effective^19,20^; however, the current service provision cannot meet demand. The panel voted unanimously to increase resources in this service in order to serve the local population effectively, as there is good evidence to support this programme.	0	0	13
Pulmonary outreach team This service helps people with COPD avoid hospital admission and achieve earlier discharge when they are admitted. A local audit has shown good evidence for the service and patients state it is a useful, effective and appreciated service.^21^ The panel felt this was a good service, with potential benefits; however, this service may not be for all patients and there is a need to be aware of differences (e.g. in terms of rural/urban levels) between areas.	1	0	12
Housing and health This is a more aspirational proposal, questioning whether the health board could form partnerships with local authorities and their housing services to use resources in a more preventative manner, given the evidence of the link between poor housing and respiratory conditions.^22^ The panel felt this was a much longer term proposal and required service integration which was not currently available. The panel concluded that this particular candidate may be a rather long-term goal and one that perhaps falls outside the scope of this PBMA task.	7	6	0
COPD local enhanced service (LES) This candidate proposes to enhance the current LESs for COPD. Though it is worth noting, there is no formal evaluation of the outcomes related to the service at present due to the introduction of the LES less than 12 months previously. The panel voted to maintain current activity as there has been no formal evaluation and no evidence of the outcomes of the service. It should also be noted two members of the panel abstained from voting on the grounds of lack of evidence.	6	5	0
Smoking cessation (Level 3 pharmacy) This candidate proposes further resources for Level 3 pharmacy smoking cessation, encompassing services within community pharmacies that offer brief interventions, advice and behavioural support, as well as supplying nicotine replacement therapy. Smoking cessation is evidenced to be a very cost-effective method to reduce morbidity and mortality from respiratory disease (in addition to other health effects).^23^ The panel felt this is a very effective and cost-effective service with potential to reach more people and build a more universal service with further resources.	1	0	12

### Marginal analysis

**Fig. 2:**
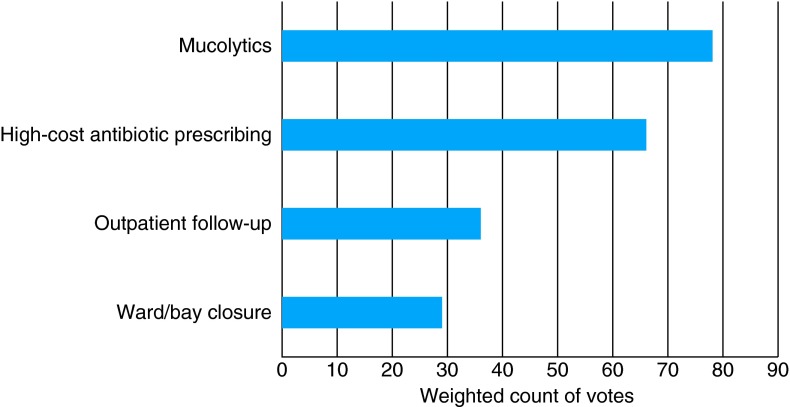
Results from the electronic ranking exercise of candidate interventions for disinvestment (higher count is higher priority).

**Fig. 3:**
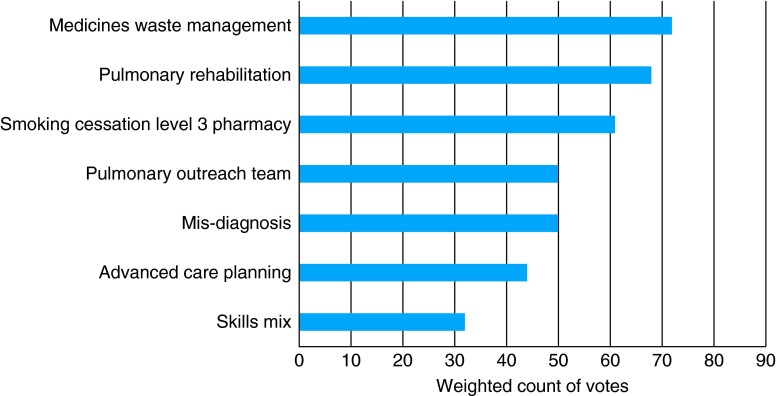
Results from the electronic ranking exercise of candidate interventions for investment (higher count is higher priority).

The panel were asked to rank in order their top priority to their lowest priority for candidates they stated they wished to invest in. The exercise was then repeated for the candidates the panel stated they wished to disinvest in. During the task, the panel prioritized mucolytics and high-cost antibiotic prescribing as areas for disinvestment, and medicine waste management and pulmonary rehabilitation for investment. The results of the disinvestment ranking exercises are presented in Fig. 2, and the results from the investment ranking exercise are presented in Fig. 3.

## Discussion

### Main findings of this study

This study demonstrates the PBMA framework can be used at a local health board level to make resource reallocation decisions. It also demonstrates the need to adapt techniques to find an appropriate way for panel members to fully engage with the process, in this case using online platforms to connect with clinicians whose time is extremely limited. The recommendations made by the panel were forwarded to the Medical Director of BCUHB as part of discussions of how to improve the respiratory care pathway. Initial progress has already begun, with communication from the Medical Director in relation to the disinvestment in mucolytics prescribing and redirection of funding options being explored, for example investment in pulmonary rehabilitation. Through informal discussion the panel stated they viewed, this PBMA exercise as a transparent, evidence-based decision-making tool for BCUHB. BCUHB saw the exercise presented here as a demonstration exercise, allowing the health board to explore the impact, usefulness and commitment regarding staff time and input required to employ a PBMA approach. Following the recommendations of the respiratory care pathway PBMA, BCUHB is considering the use for the PBMA framework across the health board.

### What is already known on this topic

The PBMA framework has been shown to work across a range of healthcare settings^6–10^ and has been applied to a national health improvement budget, containing multiple activities.^12^ This PBMA exercise, mirrored certain findings from Bolaji,^11^ recommending medicine waste management and pulmonary rehabilitation as areas for investment.

### What this study adds

This paper demonstrates how the PBMA framework can be applied successfully by a local health board at a micro-level to appraise a specific care pathway, in a challenging rural setting, with co-operation from a range of staff. Though members of the operational group had undertaken PBMA previously,^12^ this exercise required different skills and engagement techniques, with particular emphasis on communication. We would argue that undertaking this type of exercise in a rural area is perhaps more difficult than in an urban area. This is mainly attributed to geographical spread and inconvenience of travelling to face-to-face meetings. We found adapting the pro forma into a Survey Monkey questionnaire the most fruitful approach to generate candidates for resource reallocation, in particular with gaining opinions from clinicians and nurses.

For others wishing to apply PBMA in their local setting, the authors based on their experience offer the following advice, first ensure high-level sponsorship for the exercise (e.g. from directors or managers). This will help stakeholders and potential panel members engage with the exercise in the first place and may improve momentum in the form of retaining membership throughout the exercise, compared with an unsponsored exercise. Second, be flexible in how you initially engage with your panel, use all forms of communication to facilitate discussion and gather opinions. Third, endeavour to gain membership in the PBMA operational group from finance staff. Finance staff are particularly important in quantifying what changes to resource allocation could mean to the setting (e.g. health board, organization) in terms of potential cost-savings or investment required in order to effect changes. This information is vital in order to help panel members consider the concept of opportunity cost when appraising the candidates. Resource reallocation decisions will often be made against the backdrop of constrained or shrinking budgets; therefore, panel members need to understand that placing resources into one service, could mean taking resources away from another. Fourth, present evidence gathered on each of the candidates, pathway or components of a service in a consistent and accessible way. Evidence should be presented consistently, if you have used set categories to relay information for one candidate, pathway or component, then these categories should be repeated and populated for all candidates, pathways or components. If evidence is unavailable for one of the set categories, be transparent and explicitly state the evidence is not available. Finally, endeavour to gain opinions and recommendations to invest or disinvest through anonymized communication such as anonymous electronic voting handsets. This will reduce the risk of bandwagon voting. It will also allow panel members to express their own opinion without being influenced by others, especially if a panel member's line manager is also a member of the same PBMA panel.

### Limitations of this study

Clinical engagement was an issue throughout the process due to senior clinical commitments. We tried a number of strategies to reduce this limitation by alternating the location of the sessions to give equal representation to each of the three sites within the health board, provided video-conferencing facilities at every meeting and developed an online survey for invited panel members to propose ideas for resource re-allocation. Despite the best efforts of the operational group, there was no representation from primary care staff or patients at the final PBMA session when the panel were discussing and voting on the 13 proposed candidate interventions. Patient representation was an issue throughout the process and despite the best efforts of the operational group by contacting multiple patient representation groups and liaisons, we were unable to find a patient to be a panel member. The lack of primary care and patient input during discussion is a limitation of the process and could provide lessons for other health boards that plan on conducting similar PBMA exercises.

## Conclusion

The use of PBMA has significance in times of austerity when frameworks are needed to manage spending and provide a way forward. PBMA uses evidence and incorporates engagement with staff and service users on whom the recommendations from the process will ultimately effect. Against a backdrop of constrained and shrinking healthcare budgets, PBMA offers an evidence-based, transparent framework to make healthcare resource reallocation decisions. This process can be applied at a micro-level to explore a particular pathway and/or in a local setting as demonstrated by the exercise described. PBMA can also be applied at a macro-level exploring an entire service or national budget encompassing multiple activities.

## Authors’ contributions

A.J. chaired the PBMA sessions, facilitating the discussion, introducing the topics to be discussed and keeping the sessions to time. G.B., K.T. and F.J. provided clinical evidence for the proposed candidates presented to the panel and were present at the sessions to answer any specific questions related to the clinical evidence. V.V. and B.P. provided the budget information of respiratory care and financial evidence for the proposed candidate used in the PBMA exercise. V.V. and B.P. were also present at all sessions to answer any questions about the programme budget. J.M.C. and R.T.E. provided health economics expertise, and economic evidence for the evidence booklet. J.M.C. and R.T.E. provided procedural guidance on how to conduct a PBMA exercise based on their previous experiences applying the PBMA framework. J.M.C. also developed and conducted the electronic vote. G.B. and J.M.C. created the evidence pro forma and adapted the pro forma into an online questionnaire. All authors have contributed to and approved the final version of the manuscript for publication.

## Supplementary data

Supplementary Material is available at *PUBMED* online.

## Conflict of interest statement

A.J., F.J., V.V. and B.P. are employed by Betsi Cadwaladr University Health Board, though received no specific funding to conduct this PBMA exercise. G.B. and K.T. are employed by Public Health Wales, though received no specific funding to conduct this PBMA exercise. J.C. and R.T.E. are employed by Bangor University and are also consultant health economists for Public Health Wales, though received no additional funding for providing expertise to this PBMA exercise.

## Supplementary Material

Supplementary Data

## References

[1] Appleby J UK NHS: less money (but more bangs per buck). *BMJ* 2015;350:h1037. 25757982 10.1136/bmj.h1037

[2] Appleby J, Crawford R, Emmerson C. How cold will it be. Prospects for NHS funding 2011–17 www.kingsfund.org.uk/sites/files/kf/How-Cold-Will-It-Be-Prospects-NHS-funding-2011-2017-John-Appleby-Roweena_Crawford-Carl-Emmerson-The-Kings-Fund-July-2009.pdf (22 June 2015, date last accessed).

[3] Bevan G, Karanikolos M, Exley J et al The four health systems of the United Kingdom: how do they compare. http://www.nuffieldtrust.org.uk/sites/files/nuffield/publication/140411_four_countries_health_systems_summary_report.pdf (22 June 2015, date last accessed).

[4] Public Health Wales. Achieving prudent healthcare in NHS Wales. http://www.1000livesplus.wales.nhs.uk/sitesplus/documents/1011/Achieving%20prudent%20healthcare%20in%20NHS%20Wales%20paper%20Revised%20version%20%28FINAL%29.pdf (15 September 2014, date last accessed).

[5] Brambleby P, Fordham R. What is PBMA. Volume 4, Number 2 http://www.medicine.ox.ac.uk/bandolier/painres/download/whatis/pbma.pdf (15 September 2014, date last accessed).

[6] Donaldson C, Bate A, Mitton C et al Rational disinvestment. *QJM* 2010;103 (10):801–7. 20530539 10.1093/qjmed/hcq086

[7] Ratcliffe J, Donaldson C, Macphee S Programme budgeting and marginal analysis: a case study of maternity services. *J Public Health Med* 1996;18 (2):175–82. 8816315 10.1093/oxfordjournals.pubmed.a024477

[8] Mitton C, Donaldson C, Shellian B et al Priority setting in a Canadian surgical department: a case study using program budgeting and marginal analysis. *Can J Surg* 2003;46 (1):23–9. 12585790 PMC3211667

[9] Twaddle S, Walker A Programme budgeting and marginal analysis: application within programmes to assist purchasing in Greater Glasgow Health Board. *Health Policy* 1995;33 (2):91–105. 10144441 10.1016/0168-8510(95)93671-m

[10] Henderson LR, Scott A The costs of caring for stroke patients in a GP-led community hospital: an application of programme budgeting and marginal analysis. *Health Soc Care Community* 2001;9 (4):244–54. 11560740 10.1046/j.1365-2524.2001.00300.x

[11] Bolaji S. Programme budgeting marginal analysis (PBMA) for COPD PHCN Casebook. http://www.sph.nhs.uk/ebc/bhph/the-casebook-1/issue-s1/PHCN%20Casebook%20Issue%20S1-%20p1-2%20Bolaji.pdf (15 September 2014, date last accessed).

[12] Edwards RT, Charles JM, Thomas S et al A National Programme Budgeting and Marginal Analysis (PBMA) of Health Improvement Spending Across Wales: Disinvestment and Reinvestment Across the Life Course. *BMC Public Health* 2014;14:837. 25118054 10.1186/1471-2458-14-837 PMC4246570

[13] NHS Wales NHS Expenditure and Health Tool 2013. http://www2.nphs.wales.nhs.uk:8080/PubHObservatoryProjDocs.nsf/85c50756737f79ac80256f2700534ea3/53e2856fdb8d975280257c0000448494/$FILE/20131002_NEHT_v2i.pdf (1 September 2015, date last accessed).

[14] British Thoracic Society Standards of Care Committee. BTS statement on criteria for specialist referral, admission, discharge and follow-up for adults with respiratory disease. *Thorax* 2008;63:i1–i16. (23 February 2014, date last accessed). 18308973 10.1136/thx.2007.087627

[15] National Institute for Health & Care Excellence. Chronic Obstructive Pulmonary Disease Quality Standard. NICE QUALITY STANDARD 10 http://publications.nice.org.uk/chronic-obstructive-pulmonary-disease-quality-standard-qs10 (23 February 2014, date last accessed).

[16] Future Hospital Commission. Future hospital: caring for medical patients. https://www.rcplondon.ac.uk/projects/future-hospital-commission (23 February 2014, date last accessed).

[17] Puhan M, Scharplatz M, Troosters T et al Pulmonary rehabilitation following exacerbations of chronic obstructive pulmonary disease. *Cochrane Database Syst Rev* 2009;(1):CD005305. 19160250 10.1002/14651858.CD005305.pub2

[18] Seymour JM, Moore L, Jolley CJ et al Outpatient pulmonary rehabilitation following acute exacerbations of COPD. *Thorax* 2010;65 (5):423–8. 20435864 10.1136/thx.2009.124164

[19] Golmohammadi K, Jacobs P, Sin DD Economic evaluation of a community-based pulmonary rehabilitation program for chronic obstructive pulmonary disease. *Lung* 2004;182 (3):187–96. 15526757 10.1007/s00408-004-3110-2

[20] Griffiths T, Phillips C, Davies S et al Cost-effectiveness of an outpatient multi-disciplinary pulmonary rehabilitation programme. *Thorax* 2001;56 (10):779–84. 11562517 10.1136/thorax.56.10.779 PMC1745931

[21] Moreman A. Pulmonary Outreach Team Annual Report—1st August 2012–31st July 2013, BCUHB, Wrexham Maelor Hospital. Version 0c, 2013.

[22] Braubach M, Jacobs DE, Ormandy D Environmental burden of disease associated with inadequate housing, Summary Report. World Health Organization Regional Office for Europe http://www.euro.who.int/__data/assets/pdf_file/0017/145511/e95004sum.pdf (28 February 2014, date last accessed).

[23] National Institute for Health and Care Excellence. Smoking Cessation Services (NICE Public Health Guidance 10). http://www.nice.org.uk/guidance/ph10 (28 February 2014, date last accessed).

