# Supplementary material for: Use of programme budgeting and marginal analysis as a framework for resource reallocation in respiratory care in North Wales, UK
Source: J Public Health (Oxf). 2016 Oct 17;38(3):e352–61. doi: 10.1093/pubmed/fdv128 (PMC5072164; doi:10.1093/pubmed/fdv128)
Supplement: Supplementary Data [file supp_38_3_e352__index.html]

Use of programme budgeting and marginal analysis as a framework for resource reallocation in respiratory care in North Wales, UK — Supplementary Data 

# Use of programme budgeting and marginal analysis as a framework for resource reallocation in respiratory care in North Wales, UK

## Supplementary Data

Supplementary Data

- Supplementary File 1 - pdf file
- Supplementary File 2 - pdf file
