# Supplementary material for: Use of programme budgeting and marginal analysis as a framework for resource reallocation in respiratory care in North Wales, UK
Source: J Public Health (Oxf). 2016 Oct 17;38(3):e352–61. doi: 10.1093/pubmed/fdv128 (PMC5072164; doi:10.1093/pubmed/fdv128)
Supplement: Supplementary Data [file supp_fdv128_fdv128supp_file1.pdf]

GIG CYMRU NHS WALES

Bwrdd Iechyd Prifysgol
Betsi Cadwaladr
University Health Board

Respiratory Pathway PBMA Group

Investment/Disinvestment Candidates

**Title of programme/area of spend**	
Description	
Life course stage	
Spend	
Evidence	
Potential risks	
Likely impacts	
Additional notes	

Example:

**Mucolytics**	
Description	Mucolytics are prescription medicines whose primary purpose is to facilitate expectoration through reduction of sputum viscosity. They are generally prescribed for individuals with a diagnosis of COPD who have a chronic, productive cough.
Life course stage	Working age adults & older adults
Spend	Approximately £380,000 per annum.

Evidence	Mucolytics may reduce the number of exacerbations in people with chronic bronchitis or chronic obstructive pulmonary disease (COPD) by a small amount, but do not appear to cause any harm. If they do, the reduction is at most one fewer exacerbation every two years. One person in seven may avoid having an exacerbation provided they all take treatment every day for an average of 10 months. **Mucolytics have not been shown to slow the decline in lung function, nor improve quality of life.** As reduction in exacerbations seems the only potential benefit, mucolytics might be considered as a treatment option in patients with frequent exacerbations who cannot take any other therapies such as inhaled corticosteroids or long-acting bronchodilators, which have a stronger evidence base for their effectiveness.<br>It is not clear whether or not they have any effect when used as add-on treatment to other therapies used to reduce exacerbations.<br><br>*(Taken from: Poole P, Black PN, Cates CJ. Mucolytic agents for chronic bronchitis or chronic obstructive pulmonary disease. Cochrane Database of Systematic Reviews 2012, Issue 8. http://onlinelibrary.wiley.com/doi/10.1002/14651858.CD001287.pub4/pdf).*<br><br>NICE Guideline Recommendations:<br>• Mucolytic drug therapy should be considered in patients with a chronic cough productive of sputum. **Grade B**<br>• Mucolytic therapy should be continued if there is symptomatic improvement (for example, reduction in frequency of cough and sputum production). **Grade D**<br>• Do not routinely use mucolytic drugs to prevent exacerbations in people with stable COPD.<br><br>*(National Clinical Guideline Centre. (2010) Chronic obstructive pulmonary disease: management of chronic obstructive pulmonary disease in adults in primary and secondary care. London: National Clinical Guideline Centre. http://guidance.nice.org.uk/CG101/Guidance/pdf/English)*

Recommendation	**Disinvestment**<br>Routine prescribing of mucolytics is not recommended for people with COPD. Where indicated (as per the evidence), the impact after a 4-week trial (as per BNF prescribing guidance) should be assessed and treatment stopped if no improvement.<br>Better adherence to the recommendations and monitoring of prescribing could reduce prescribing costs.<br><br>Additionally it may be worth re-assessing current users of mucolytics, to gauge effectiveness – those that have been on mucolytics for over 3 months should either have their treatment stepped-down or discontinued.
Potential risks	• Increased risk of exacerbations for those whose treatment is effective but reduced/discontinued leading to unscheduled admissions.
Likely impacts	• Reduction in prescribing costs.<br>• Reduced waste from ineffective medicines.<br>• Improved clinical governance through development of robust prescribing guidelines.
Additional notes	Unlikely to need any investment to achieve as can be done through existing medicines governance arrangements (e.g. by GP/outpatient reviews or through COPD LES).
