# Supplementary material for: Use of programme budgeting and marginal analysis as a framework for resource reallocation in respiratory care in North Wales, UK
Source: J Public Health (Oxf). 2016 Oct 17;38(3):e352–61. doi: 10.1093/pubmed/fdv128 (PMC5072164; doi:10.1093/pubmed/fdv128)
Supplement: Supplementary Data [file supp_fdv128_fdv128supp_file2.pdf]

• High Cost Antibiotic Prescribing

<table>
<tr><td>Description</td><td>Intravenous antibiotics are a vital component of treatment for people with severe Community Acquired Pneumonia (CAP).
The Health Board has an established set of guidelines for prescribing antibiotics to treat CAP. These recommend that patients with less severe disease receive oral antibiotic treatment, with intravenous therapy reserved for those with more severe disease.
There is evidence to suggest that although the prescribing guidelines are agreed, these are not always adhered to, with many patients receiving IV therapy when this is not indicated.</td></tr>
<tr><td>Life course stage</td><td>Working age adults & older adults</td></tr>
<tr><td>Spend</td><td>TBC

(NB: See below for costs of antibiotics)</td></tr>
<tr><td>Evidence</td><td>Current prescribing guidelines recommend that intravenous antibiotics are prescribed for individuals who present with community acquired pneumonia and a CURB-65 score of at least 3, or are clinically considered a severe case. Individuals with a lower score should be commenced on oral treatment only.

In addition, any patient in whom IV antibiotics are prescribed should have this prescription reviewed after 48 hours, and where there are no signs of sepsis, be stepped down to oral antibiotics.

These guidelines are hospital policy and based on existing evidence.</td></tr>
<tr><td>Potential risks</td><td>Effective treatment not being started early
Policy rather than clinical judgement dictating treatment.</td></tr>
<tr><td>Likely impacts</td><td>Improved patient care through clinically appropriate prescribing.
Reduced medicines waste.</td></tr>
<tr><td>Additional notes</td><td>
<table>
<tr><th>CURB-65 Score</th><th>Treatment</th><th>Cost per day</th></tr>
<tr><td>0-1</td><td>Amoxicillin 500mg PO TDS</td><td>24 pence</td></tr>
<tr><td>2</td><td>Amoxicillin 500mg PO TDS, and Clarithromycin 500mg PO BD</td><td>74 pence</td></tr>
<tr><td>3+</td><td>Amoxicillin 1g IV TDS, and Clarithromycin 500mg PO/IV BD</td><td>(Clarith. PO) £3.80
(Clarith. IV) £22.20</td></tr>
<tr><td>3+ (high-risk hx)</td><td>Tazocin 4.5g IV TDS, and Clarithromycin 500mg PO/IV BD</td><td>(Clarith. PO) £46.01
(Clarith. IV) £64.41</td></tr>
</table>
</td></tr>
</table>

CURB-65 Score	Treatment (PENICILLIN AVOID)	Cost per day
0-2	Doxycycline 200mg PO stat, then 100mg PO OD.	(day 1) 28 pence (day 2+) 14 pence
3+ (if no penicillin anaphylaxis)	Cefuroxime 1.5g IV TDS, and Calrithromycin 500mg PO/IV BD	(Clarith. PO) £15.65 (Clarith. IV) £34.05
3+ (Central & West)	Ciprofloxacin 500mg PO BD, and Vancomycin 1g IV BD	£26.20
3+ (East)	Ciprofloxacin 500mg PO BD, and Teicoplanin 600mg IV BD	£19.56

	Dose	BNF cost (generic)	Cost per dose	Daily cost
Tazocin IV	4.5g, tds	£15.17 per 4.5g vial	£15.17	£45.51
Amoxicillin IV	1g, tds	£1.10 per 1g vial	£1.10	£3.30
Amoxicillin PO	500mg, tds	£1.64 for 21 capsules (500mg)	£0.08	£0.24
Clarithromycin IV	500mg, bd	£9.45 per 500mg vial	£9.45	£18.90
Clarithromycin PO	500mg, bd	£3.47 for 14 tabs (500mg)	£0.25	£0.50
Doxycycline PO	100mg od	£1.08 per 8 caps	£0.14	£0.14
Cefuroxime IV	1.5g, tds	£5.05 per 1.5g vial	£5.05	£15.15
Ciprofloxacin PO	500mg BD	£1.07 for 10 tabs (500mg)	£0.11	£0.22
Vancomycin IV	1g BD*	£12.99 per 1g vial	£12.99	£25.98
Teicoplanin IV	600mg BD†	£3.57 per 200mg vial, £6.10 per 400mg vial	£9.67	£19.34

* Assumed IV administration, and 1g BD
† For three doses, then once daily.

• Pulmonary Rehabilitation Programme

Description	Pulmonary Rehabilitation is an evidenced based, multidisciplinary, multi-component programme of care that helps people with Chronic Obstructive Pulmonary disease to better manage their symptoms.
Life course stage	Working age & older adults
Spend	Enhancing the current service will require approximately £283k of additional funding per year. Some of this has already been realised through efficiency savings, leaving a shortfall of approximately £220k that is needed.
Evidence	The evidence for pulmonary rehabilitation shows that it:<br>• reduce mortality from COPD[1]<br>• reduce hospital admissions[1]<br>• reduce inpatient hospital days[2]<br>• reduce readmission rates (e.g. from 33 – 7%)[3]<br>• reduce the number of GP home visits[2]<br>• improve health-related quality of life in patients with COPD after suffering an exacerbation (e.g. dyspnoea, fatigue, depression, and patient control of the disease)[1,4-9]<br>• be highly cost-effective – it is substantially below the NICE threshold for cost-effectiveness, at only £2,000 - £8,000 per QALY[2]<br>• be cost-saving[2, 10] - one study showed an overall cost saving of £152 per patient per pulmonary rehabilitation programme[2]<br><br>In addition, referral into a pulmonary rehabilitation programme is now included as part of the QOF for general practices in North Wales.
Potential risks	• Investment required to enhance the service.
Likely impacts	• Realignment of service to better meet the needs of local population<br>• Increased service capacity with reduced waiting times<br>• Improved health outcomes<br>• Reduced in unscheduled admissions and readmissions as a result of COPD.

Additional notes	A comprehensive business case has already been produced that outlines the expected benefit and full costs of the service.

References:

1. Puhan, M. et al. Pulmonary rehabilitation following exacerbations of chronic obstructive pulmonary disease *Cochrane Database Syst Rev*; 2009;(1):CD005305
2. Griffiths et al. (2001) “Cost-effectiveness of an outpatient multi-disciplinary pulmonary rehabilitation programme” Thorax 56: 779 – 784
3. Outpatient pulmonary rehabilitation following acute exacerbations of COPD. Seymour JM et al. Thorax 2010 May;65(5):423-8
4. Devine EC, Pearcy J. Meta-analysis of the effects of psycho educational care in adults with chronic obstructive pulmonary disease. Patient Educ Couns 1996; 29:167–178/ Wempe JB, Wijkstra PJ. The influence of rehabilitation on behaviour modification in COPD. Patient Educ Couns 2004; 52:237–241
5. Liesker JJ, Postma DS, Beukema RJ, et al. Cognitive performance in patients with COPD. Respir Med 2004;98:351–356
6. Griffiths TL, Burr ML, Campbell IA, et al. Results at 1 year outpatient multidisciplinary pulmonary rehabilitation: a randomised controlled trial. Lancet 2000; 355:362–368
7. Emery CF, Hauck ER, Schein RL, et al. Psychological and cognitive outcomes of a randomized trial of exercise among patients with chronic obstructive pulmonary disease. Health Psychol 1998; 17:232–240
8. Pulmonary rehabilitation: Joint ACCP/AACVPR evidence-based clinical practice guidelines-recommendation 7
9. Ries AL, Kaplan RM, Myers R, et al. Maintenance after pulmonary rehabilitation in chronic lung disease: a randomized trial. Am J Respir Crit Care Med 2003; 167:880–888
10. Derom et al. (2007) “Literature Review – Pulmonary Rehabilitation in chronic obstructive pulmonary disease” Annales de réadaptation et de médecine physique 50: 615–626 Golmohammadi, et al. (2004) “Economic Evaluation of a Community-Based Pulmonary Rehabilitation Program for Chronic Obstructive Pulmonary Disease”, Lung 182:187 - 196
